# Acute Cade Oil Poisoning in Children: Report of Two Cases

**DOI:** 10.7759/cureus.89717

**Published:** 2025-08-10

**Authors:** Mohammed Amine Oulalite, Samia Berrichi, Safaa Kachmar, Ilyass Laaribi, Houssam Bkiyar

**Affiliations:** 1 Department of Anesthesia and Reanimation, University Hospital Mohammed VI, Faculty of Medicine and Pharmacy, Mohammed First University, Oujda, MAR

**Keywords:** cade oil poisoning, pediatric intoxication, phenol toxicity, topical exposure, traditional medicine

## Abstract

Cade oil, widely used in traditional Moroccan medicine for its perceived therapeutic properties, can lead to severe toxicity when ingested or applied extensively to the skin, particularly in infants and young children. We report two cases of toddlers who developed altered consciousness following widespread topical application. Laboratory findings revealed mild hepatic cytolysis and inflammatory markers, though imaging and cerebrospinal fluid analysis were normal. Both patients responded well to supportive care, including thorough decontamination and close clinical monitoring, and made a full recovery. These cases highlight the potential dangers of unregulated traditional treatments and underscore the need for greater public awareness, early clinical recognition, and appropriate medical intervention to prevent life-threatening complications.

## Introduction

Cade oil, also known as tar oil of *Juniperus oxycedrus* or “qtran rqiq” in Morocco, is an essential oil commonly used in traditional Moroccan medicine [1]. In folk practice, it serves as a parasiticide and antiseptic for skin conditions such as scabies, alopecia, pityriasis, seborrhea, and eczema. Toxicity usually results from ingesting large amounts or from prolonged, extensive topical application [2]. This report highlights a potentially fatal form of intoxication that, while relatively rare, poses a serious risk if not promptly recognized. To illustrate this, we describe two cases of cade oil poisoning in children admitted to our pediatric emergency department.

## Case presentation

We report two cases of cade oil poisoning in children aged two and three years, both previously healthy, who were admitted to the pediatric emergency department with altered consciousness lasting about two hours. Neither child had a fever. Upon arrival, Glasgow Coma Scale scores were 11 and 13, respectively, with no focal neurological deficits noted. Both were hemodynamically and respiratory stable. According to family members, cade oil had been applied extensively over their entire bodies for 1-3 hours daily over the past 8-10 weeks for perceived therapeutic benefits.

Laboratory evaluation showed mild inflammatory changes, including elevated C-reactive protein, leukocytosis, and hepatic cytolysis. Blood glucose was normal, and no other metabolic disturbances were detected (Table 1). Cerebrospinal fluid analysis and brain CT scans were unremarkable in both cases. With different causes excluded, cade oil exposure was identified as the most likely cause of their presentation.

**Table 1:** Lab results of the two patients. ALAT/ALT: alanine aminotransferase (liver enzyme), ASAT/AST: aspartate aminotransferase (liver enzyme), LDH: lactate dehydrogenase (marker of tissue damage), ALP: alkaline phosphatase (liver/bone enzyme), CRP: C-reactive protein (marker of inflammation), GCS: Glasgow Coma Scale.

Parameters	Normal range	Patient 1	Patient 2
Age (years)		2	3
GCS	3–15	11	13
Hemoglobin (g/dL)	12–17	14.2	13.8
White blood cell count (×10⁹/L)	4.0–11.0	7.5	8.1
Blood glucose (mg/dL)	70–110	92	105
Total bilirubin (mg/dL)	0.3–1.2	0.8	1.1
Direct bilirubin (mg/dL)	0.1–0.4	0.3	0.4
ALAT/ALT (U/L)	<40	58 ↑	61 ↑
ASAT/AST (U/L)	<40	50 ↑	54 ↑
LDH (U/L)	140–280	310 ↑	298 ↑
ALP (U/L)	40–130	145 ↑	138 ↑
CRP (mg/L)	<5	12 ↑	10 ↑

Both patients underwent thorough skin cleansing and received supportive treatment with close monitoring. Their condition improved steadily, with complete resolution of neurological symptoms. N-acetylcysteine was not given, as both showed a favorable clinical course. After four days of hospitalization, they were discharged in stable condition.

## Discussion

Cade oil, also known in the Moroccan dialect as “qtran rqiq,” is obtained by dry distillation of the branches of Juniperus oxycedrus. It is widely used in traditional Moroccan medicine for its analgesic, digestive, bronchopulmonary, keratolytic, antipruritic, antiseptic, and antiparasitic properties [1]. Readily available without a prescription, it is applied topically for skin conditions such as psoriasis and scabies, incorporated into cosmetic products, and occasionally taken orally as a deworming agent. While it offers certain therapeutic benefits, including anti-inflammatory effects, it also carries a significant risk of poisoning due to its phenol content. Phenol is rapidly absorbed and metabolized in the liver, producing toxic free radicals that can cause multi-organ injury [3,4].

Cade oil intoxication can affect the cardiovascular, neurological, renal, and respiratory systems. Phenol, its most toxic component, is quickly hydroxylated in the liver to semi-quinone radicals. When exposure exceeds the liver’s conjugation capacity, these radicals generate free radicals capable of causing hepatic and renal injury [2,3]. Hepatic cytolysis from centrilobular necrosis has been documented in several phenol poisonings [4]. Elevated aminotransferases and lactate dehydrogenase (LDH) have been reported after ingestion of phenol-containing substances, though levels generally normalize over time. In our patient, only mild hepatic enzyme elevation was observed.

Previous studies have reported elevated liver enzymes, such as aminotransferases and lactate dehydrogenase (LDH), following ingestion of phenol-containing substances, indicating hepatic injury. However, enzyme levels typically normalize over time. In our patient, only a mild elevation of hepatic enzymes was noted. Pulmonary complications range from pneumonitis to acute pulmonary edema (APE) after both topical and oral exposure. Wu et al. reported pneumonia in a 44-year-old man following ingestion of 300 mL of cresol solution [5]. Rahmani et al. described APE in a four-month-old infant after cade oil administration [6], while Achour et al. reported the death of a 40-day-old infant from APE following facial and scalp application of cade oil [7].

Neurological effects involve both the central and peripheral nervous systems and include involuntary movements, seizures, dizziness, hypotonia, headaches, and myoclonic coma [8,9]. Infants are particularly vulnerable due to their higher surface-area-to-body-weight ratio and immature hepatic detoxification systems, which limit phenol metabolism and elimination [10].

Management of cade oil poisoning involves hospitalizing symptomatic patients in an intensive care setting for close monitoring and rapid intervention. For dermal exposure, immediate and thorough washing with soapy water is essential to reduce absorption. Polyethylene glycol may also be applied to bind phenol and further limit uptake [11]. Supportive care includes intravenous hydration to maintain hemodynamic stability and correct acid-base imbalances. To prevent hepatic injury, N-acetylcysteine (NAC) is recommended for its glutathione-restoring and antioxidant effects, typically given as a loading dose of 150 mg/kg followed by 60 mg/kg every four hours [12]. In our two cases, thorough skin cleansing and close monitoring alone were sufficient for full recovery.

## Conclusions

Cade oil remains widely available and frequently used in traditional medicine, particularly in Morocco. Although often considered harmless, its phenol content can cause life-threatening toxicity, especially in young children. Our report of two pediatric cases of cade oil intoxication underscores the importance of early diagnosis and supportive care. The favorable outcomes in these cases were due to prompt intervention, including decontamination and close monitoring. These cases highlight the dangers of unsupervised use of traditional remedies and the urgent need for public education and preventive measures to reduce such poisonings in children.
